# More Anterior *in vivo* Contact Position in Patients With Fixed-Bearing Unicompartmental Knee Arthroplasty During Daily Activities Than *in vitro* Wear Simulator

**DOI:** 10.3389/fbioe.2021.666435

**Published:** 2021-05-20

**Authors:** Huiyong Dai, Nan Zheng, Diyang Zou, Zhemin Zhu, Ming Han Lincoln Liow, Tsung-Yuan Tsai, Qi Wang

**Affiliations:** ^1^Department of Orthopedics, Shanghai Jiao Tong University Affiliated Sixth People’s Hospital, Shanghai, China; ^2^School of Biomedical Engineering and Med-X Research Institute, Shanghai Jiao Tong University, Shanghai, China; ^3^Engineering Research Center of Digital Medicine and Clinical Translation, Ministry of Education, Shanghai, China; ^4^Shanghai Key Laboratory of Orthopaedic Implants and Clinical Translation R&D Center of 3D Printing Technology, Department of Orthopaedic Surgery, Shanghai Ninth People’s Hospital, Shanghai Jiao Tong University School of Medicine, Shanghai, China; ^5^Department of Orthopaedic Surgery, Singapore General Hospital, Singapore, Singapore

**Keywords:** fluoroscopy, 2D-to-3D registration, unicompartmental knee arthroplasty, contact position, *in vivo*, biomechanics

## Abstract

**Background:**

While *in vitro* wear simulation of unicompartmental knee arthroplasty (UKA) showed outstanding long-term wear performance, studies reported that polyethylene (PE) wear was responsible for 12% fixed-bearing (FB) UKA failure. This paper aimed to quantify the *in vivo* 6-degrees-of-freedom (6-DOF) knee kinematics and contact positions of FB UKA during daily activities and compare with the previous results of *in vitro* wear simulator.

**Methods:**

Fourteen patients following unilateral medial FB UKA received a CT scan and dual fluoroscopic imaging during level walking, single-leg deep lunge, and sit-to-stand motion for evaluating *in vivo* 6-DOF FB UKA kinematics. The closest point between surface models of the femoral condyle and PE insert was determined to locate the medial compartmental articular contact positions, which were normalized relative to the PE insert length. The *in vivo* contact area was compared with the *in vitro* wear region in previous simulator studies.

**Results:**

The *in vivo* contact positions during daily activities were more anterior than those in the previous *in vitro* wear simulator studies (*p* < 0.001). Significant differences in the femoral anteroposterior translation and tibial internal rotation during the stance phase were observed and compared with those in lunge and sit-to-stand motions (*p* < 0.05). The *in vivo* contact position located anteriorly and medially by 5.2 ± 2.7 and 1.8 ± 1.6 mm on average for the stance phase, 1.0 ± 2.4 and 0.9 ± 1.5 mm for the lunge, and 2.1 ± 3.3 and 1.4 ± 1.4 mm for sit-to-stand motion. The *in vivo* contact position was in the more anterior part during the stance phase (*p* < 0.05).

**Conclusion:**

The current study revealed that the contact position of FB UKA was located anteriorly and medially on the PE insert during *in vivo* weight-bearing activities and different from previous findings of the *in vitro* wear simulator. We should take *in vivo* 6-DOF knee kinematics and contact patterns of FB UKA into account to reproduce realistic wear performance for *in vitro* wear simulator and to improve implant design.

## Introduction

Unicompartmental knee arthroplasty (UKA) is an increasingly popular surgical treatment for end-stage unicompartmental osteoarthritis. Advantages of UKA in comparison with total knee arthroplasty (TKA) have been reported, including smaller surgical incision, less blood loss (Rajasekhar et al., 2004), shorter hospital stays and quicker recovery (Lombardi et al., 2009), better functional outcomes (Beard et al., 2017), reduced awareness of an artificial joint (Zuiderbaan et al., 2017), and better restoration of knee kinematics (Patil et al., 2005). However, several complications, including aseptic loosening, osteoarthritis progression, undesirable polyethylene (PE) wears, and particle-induced osteolysis (List et al., 2016), have contributed to a significantly lower survival rate in UKA than TKA (Liddle et al., 2014). Two design types are available in UKA systems, i.e., fixed-bearing (FB) and mobile-bearing (MB). The FB UKA has survival rates, clinical outcomes, and patient satisfaction similar to the MB design (Neufeld et al., 2018; Pronk et al., 2020). However, PE wear was shown to result in 12% of FB UKA failure (List et al., 2016). The UKA has been extended to younger patients who suffer from unicompartmental osteoarthritis to restore the normal level of physical activity (Calkins et al., 2021), indicating higher demand for long-term survival of UKA. Thus, it is necessary to investigate the FB UKA’s contact mechanisms to reduce PE wear.

Retrieved PE inserts of UKA with severe wear were characterized by delamination, deformation, peripheral cracking, pitting, and abrasion (Bartley et al., 1994). However, only burnishing and pitting on PE surface can be observed for *in vitro* knee wear simulator under standard level walking loading (Harman et al., 2010; Sutton et al., 2010), which is inconsistent with *in vivo* conditions and clinical outcomes of FB UKA. This discrepancy is likely due to the unrealistic boundary conditions for the *in vitro* knee wear simulator of FB UKA. The widely used boundary conditions of FB UKA include loading and displacement during level walking specified in ISO 14243-1:2009(E), which are measured in TKA patients (Lu et al., 1998; Taylor et al., 1998). However, the knee kinematics in ISO 14243-1:2009(E) are significantly different from the *in vivo* situation for UKA patients during level walking (Jones et al., 2016). Besides, the abrasive-adhesive wear and delamination mechanisms of FB UKA can be better reproduced under highly demanding activities than single-level walking loading (Schwiesau et al., 2013a,b), illustrating the importance of other functional activity conditions in UKA wear simulation. These differences may contribute to the inconsistent wear results observed between *in vivo* implant retrieval analysis and the *in vitro* knee wear simulations (Bartley et al., 1994; Harman et al., 2010; Sutton et al., 2010).

Additional research regarding *in vivo* FB UKA kinematics is necessary to understand better the wear mechanisms of UKA components (Schmalzried and Callaghan, 1999). Fluoroscopic tracking and the EOS system (EOS Imaging, SA, French) have been used to quantify the *in vivo* UKA kinematics and articular contact (Mochizuki et al., 2013; Tsai et al., 2016b). The precise and detailed knee kinematics and kinetics of UKA during weight-bearing daily activities can give significant insights into the *in vitro* UKA simulator’s loading condition when evaluating its wear performance. The knee flexion, tibial internal rotation, and femoral anteroposterior movement of FB UKA during several activities have been reported (Mochizuki et al., 2013; Zumbrunn et al., 2020). However, other kinematic parameters were not reported, e.g., knee abduction-adduction; and more demanding daily activities should be investigated. Besides, the contact kinematics of TKA during functional activities, such as level walking, single-leg lunge, and sit-to-stand motion, are evaluated for the prediction of the wear performance (Argenson et al., 2002; Varadarajan et al., 2008; Catani et al., 2010). However, no prior study investigated the *in vivo* contact position during functional activities for patients following FB UKA. Thus, it is essential to investigate *in vivo* 6-degrees-of-freedom (6-DOF) knee kinematics and contact pattern following FB UKA during functional activities to predict better the wear performance and service life of FB UKA.

The purposes of the current study were (1) to quantify the *in vivo* 6-DOF knee kinematics and contact positions of FB UKA during the walking, lunge, and sit-to-stand activities; (2) to compare its kinematics and contact patterns among different functional activities; and (3) to compare *in vivo* contact kinematics with previous findings of *in vitro* wear simulator. We hypothesized that kinematics and contact patterns of FB UKA varied among walking, lunge, and sit-to-stand activities and that *in vivo* contact kinematics were significantly different from those of the *in vitro* wear simulator.

## Materials and Methods

### Patient Demographic Data

The study protocol was approved by the Ethics Committee of Shanghai Sixth People’s Hospital, China (No. 2017-084). Fourteen patients (three males and 11 females) who received unilateral medial UKA implantation (six left and eight right knees) participated in this study. The inclusion criteria were as follows: (1) unilateral medial compartmental end-stage osteoarthritis; (2) aging from 18 to 80 years; and (3) signed the informed consent before participation in the study. The exclusion criteria were as follows: (1) any postoperative complications or musculoskeletal diseases 6 months after surgery; (2) severe neurological deficit or symptoms; (3) severe cardiovascular and cerebrovascular diseases; and (4) pregnancy or breastfeeding. All patients underwent Restoris MCK FB UKA (Stryker, MI, United States) using Mako robotic system (Stryker, MI, United States) with cobalt–chromium femoral condyle, highly cross-linked PE inserts with low congruence geometrical design, and titanium tibial baseplate. The higher component positioning accuracy during Mako robotic-assisted UKA implantation has been verified than traditional manual techniques (Bell et al., 2016). All patients participated in this study 6 months after unilateral UKA surgery. The average age of the patients was 63.9 years (±6.6, range 52 to 72); the average weight, height, and body mass index (BMI) were 69.6 kg (±10.9, range 51.2 to 97.2), 159.0 cm (±8.5, range 148.6 to 178.2), and 27.4 kg/m^2^ (±2.8, range 22.6 to 31.7), respectively. The average follow-up period was 7.1 months (±1.2, range 6.0 to 10.0).

### CT-Based 3D Reconstruction of the Knee

**FIGURE 1:**
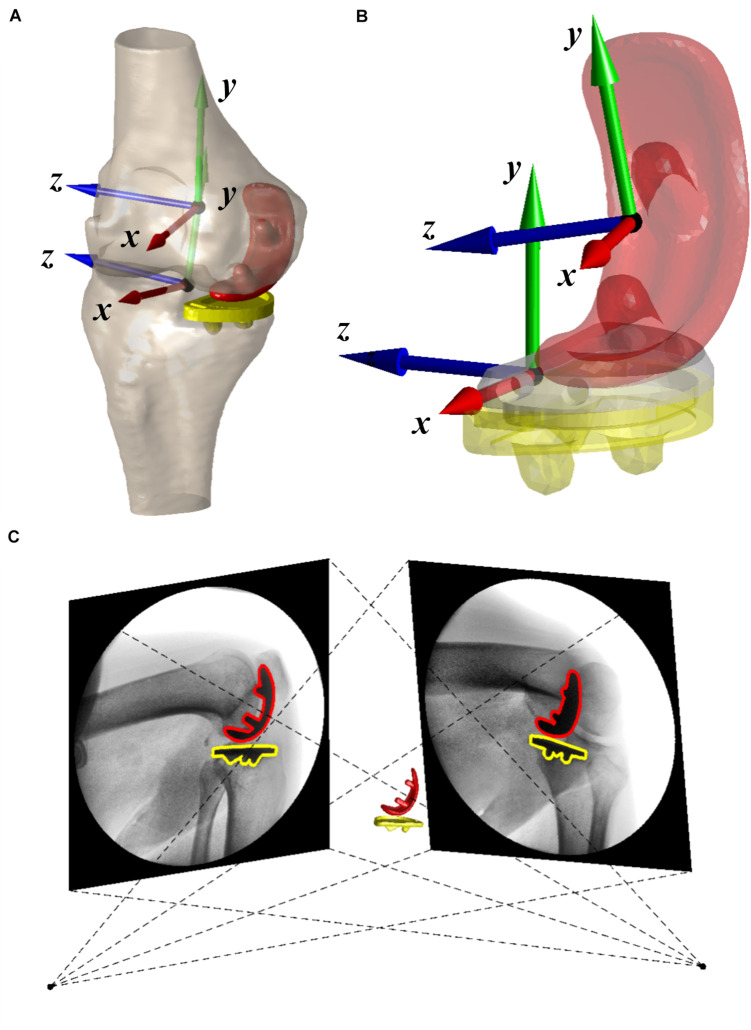
**(A)** Three-dimensional surface models of the operated knee with coordinate systems and fixed-bearing unicompartmental knee arthroplasty (UKA). **(B)** The coordinate systems of UKA components. **(C)** Virtual environment of dual fluoroscopic imaging system (DFIS) was shown. The positions of red and yellow UKA components were adjusted until it matched the projection of 3D computer-aided design (CAD) models with outlines in two fluoroscopic images.

All patients underwent a 64-slice computed tomography (CT) scan (Sensation 64, Siemens, Germany) in the supine position for approximately 100 mm above and below the joint line of the knee, as well as the hip and ankle for preoperative planning. CT scan was performed again for the implanted knee 6 months after surgery. The bones and implants were segmented from CT images using a region-grow method in Amira 6.7.0 (Thermo Fisher Scientific, Rockford IL, United States) to reconstruct the 3D surface models of UKA components (femoral condyle, and tibial baseplate) and bones (proximal femoral head, distal femur, tibial plateau, and ankle). Anatomical coordinate systems of the femur and tibia were created according to bony landmarks (Grood and Suntay, 1983). The preoperative models were then aligned to postoperative femur and tibia using iterative closest points (Figure 1A; Besl and McKay, 1992; Tsai et al., 2013) to determine the coordination system of the operated knee. The residual bones were involved in the aligning method except for the medial compartment. A 3D deviation analysis on postoperative and aligned preoperative models indicated that the average ± standard deviation (SD) of difference was 0.28 ± 0.05 mm for the femur and 0.32 ± 0.08 mm for the tibia. The coordinate systems of UKA components were built according to the geometric features that the manufacturer provided in the 3D computer-aided design (CAD) model (Figure 1B). The 3D CAD models of implants were then aligned with reconstructed implants to determine the position of implants with respect to the femur and tibia for contact analysis (Besl and McKay, 1992). The average ± SD of distances between 3D CAD and reconstructed implant models were 0.27 ± 0.06 mm for the femoral condyle and 0.33 ± 0.07 mm for the tibial baseplate.

### Dual Fluoroscopic Imaging System

The dual fluoroscopic imaging system (DFIS) comprises two mobile fluoroscopes (BV Pulsera, Philips Medical, Netherlands) set in an approximately orthogonal position. The knee of each patient was under the surveillance of DFIS (30s snapshots per second with an 8-ms pulse width) as the patient performed functional activities, including level walking on a treadmill at a self-selected speed, single-leg deep lunge, and sit-to-stand motion. The average and SD of the walking velocity of all unilateral UKA patients was 0.6 ± 0.1 m/s. The height of the chair was adapted for each patient to ensure that patients performed sit-to-stand motion with the same knee flexion angle during the test. Then, the series of 2D dynamic fluoroscopic images and 3D CAD UKA component models of the knee were imported into a customized virtual DFIS system built-in MATLAB (MATLAB, MathWorks, Natick, MA, United States). The accurate positions of each component were acquired independently by matching the projection of the 3D CAD model with the outline of fluoroscopic images (Figure 1C). The DFIS tracking technique was evaluated with 0.27° and 0.10-mm accuracy for femoral condyle and 0.39° and 0.18-mm accuracy for tibial baseplate (Tsai et al., 2016a). The femur’s and tibia’s spatial positions were determined based on the component models in a virtual DFIS environment. The flexion–extension, adduction–abduction, and internal–external rotation of the tibia relative to the femur and anterior–posterior, proximal–distal, and medial–lateral translations of the femur relative to the tibia were calculated (Grood and Suntay, 1983). The 6-DOF of FB UKA was quantified along with the weight-bearing stance phase during gait, lunge, and sit-to-stand motion.

### *In vivo* Medial Articular Contact Measurements

The coordinate systems of UKA components were built to evaluate the *in vivo* medial articular contact pattern during different functional activities. The spatial contact positions on the PE insert and the medial articular surface were determined by tracking the closest point between surfaces of the femoral components and PE insert (Glynjones et al., 2008; Teeter et al., 2018). The coordinates of the contact points were transformed with respect to the centroid of the PE insert, and anteroposterior and mediolateral axes of the tibial component. Also, the length of the PE insert was utilized to normalize the contact positions. To evaluate the sensitivity of contact measurement, we added the random Gaussian noises in agreement with the DFIS tracking error to 6-DOF of femoral and tibial components (Tsai et al., 2016a). The average ± SD of contact location error was 0.16 ± 0.39 mm in the anteroposterior direction and 0.14 ± 0.24 mm in the mediolateral direction. Each patient’s contact trajectories were mapped onto a representative PE insert with 43-mm length to show the average contact pattern. To explain the difference between measured *in vivo* contact positions of FB UKA during functional activities and previous *in vitro* wear region, the centers of wear region or stress distribution area on superior surface of PE inserts were quantified and mapped onto the current representative PE insert (Kwon et al., 2013; Schroeder et al., 2013; Schwiesau et al., 2013b; Koh et al., 2019).

### Statistical Analysis

Average and SD of all measurements were reported relative to gait cycle in level walking and relative to the knee flexion angle in deep lunge and sit-to-stand motions. The average and range of contact positions during different functional activities were tested using the Kolmogorov–Smirnov test for normality. Considering that *in vitro* wear performance varied under different activity loadings (Schwiesau et al., 2013b), the one-way ANOVA was used to analyze the significance of differences among motions followed by the Duncan *post hoc* test. The Wilcox rank sum test was performed to explain the differences between *in vivo* contact positions and previous *in vitro* wear regions. The level of significance was set as 0.05. A *post hoc* statistical power analysis was performed to examine the differences among motions (G^∗^Power 3.1.9.7^1^).

## Results

### *In vivo* 6-Degrees-of-Freedom Knee Kinematics and Contact Positions

The kinematics of the stance phase showed a different pattern from lunge and sit-to-stand motions, and knee flexion–extension and femoral anteroposterior translation have main DOFs during the stance phase with limited variation in other DOFs (Supplementary Figure 1). Significant femoral posterior movement and tibial internal rotation were observed with respect to the increase of knee flexion angle for lunge and sit-to-stand motions (*p* < 0.05; Supplementary Figures 2,3). Detailed *in vivo* 6-DOF of FB UKA during the stance phase of the gait cycle, single-leg deep lunge, and sit-to-stand motions are shown in Supplementary Figures 1–3.

**FIGURE 2:**
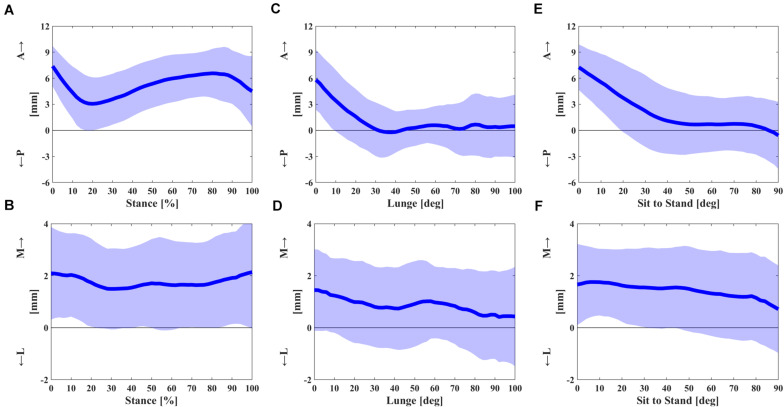
**(A,B)** The anterior–posterior and medial–lateral contact excursion during the stance phase of gait cycle. **(C,D)** The anterior–posterior and medial–lateral contact excursion relative to knee flexion angle in single-leg lunge. **(E,F)** The anterior–posterior and medial–lateral contact excursion relative to knee flexion angle in sit-to-stand motion. The dashed areas represent one standard deviation (SD).

A curve pattern with two inflections was observed in an anteroposterior contact excursion during the stance of the gait cycle (Figure 2A). The contact positions moved posteriorly from 7.4 ± 2.3 mm at the beginning to 3.1 ± 3.1 mm at 20% of the stance phase and then moved anteriorly to 6.6 ± 2.8 mm at 80% of the stance phase and moved posteriorly again to 4.5 ± 4.1 mm in the end (Figure 2A). The average and range of contact positions in anteroposterior direction during the stance phase were 5.2 ± 2.7 and 6.0 ± 1.6 mm during the swing phase, respectively (Table 1). There was a little mediolateral contact excursion during stance, with an average of 1.8 ± 1.6 mm and a range of 1.9 ± 0.6 mm (Figure 2B and Table 1).

**TABLE 1:** *In vivo* articular contact excursion during stance phase, single-leg lunge, and sit-to-stand motion and *in vitro* wear region center in anterior–posterior and medial–lateral directions.

**Activities**	**Anterior–posterior**				**Medial–lateral**			
	**Average, mm**	**Normalized, %**	**Range, mm**	**Normalized, %**	**Average, mm**	**Normalized, %**	**Range, mm**	**Normalized, %**
Stance	5.2 ± 2.7	12.0 ± 6.2	6.0 ± 1.6	13.9 ± 3.7	1.8 ± 1.6	6.8 ± 6.2	1.9 ± 0.6	7.5 ± 2.3
Lunge	1.0 ± 2.4	2.4 ± 5.5	7.9 ± 2.7	18.5 ± 6.4	0.9 ± 1.5	3.3 ± 5.6	2.4 ± 1.3	9.2 ± 5.2
Sit-to-stand	2.1 ± 3.3	4.9 ± 7.6	8.6 ± 2.6	19.9 ± 6.0	1.4 ± 1.4	5.5 ± 5.5	2.2 ± 1.2	8.3 ± 4.5
*In vitro*	−0.5 ± 1.0	−1.1 ± 2.2			0.7 ± 0.6	2.6 ± 2.3		

*The *in vitro* wear region centers were calculated from data in Kwon et al. (2013), Schroeder et al. (2013), Schwiesau et al. (2013b), and Koh et al. (2019). Data are given as average ± standard deviation.*

Significant posterior contact gliding of FB UKA was noticed in early flexion during the deep lunge. The articular contact of the UKA during flexion showed a posterior excursion from 5.8 ± 3.5 mm at knee flexion 0° to −0.2 ± 2.8 mm at 36° and remained almost unchanged in mid and deep flexion (Figure 2C). The contact excursion in mediolateral direction showed the average and range of 0.9 ± 1.5 and 2.4 ± 1.3 mm (Figure 2D and Table 1).

The posterior contact gliding relative to early flexion in sit-to-stand motion was also observed with a similar pattern to deep lunge. The anteroposterior contact position moved posteriorly from 7.3 ± 2.6 mm at knee flexion 0° to 0.9 ± 3.7 mm at 44°, remained nearly the same in mid flexion, and moved posteriorly from 0.7 ± 3.3 mm at 76° to −0.6 ± 3.9 mm at 90° (Figure 2E). The average and range of a mediolateral contact excursion were 1.4 ± 1.4 and 2.2 ± 1.2 mm, respectively (Figure 2F and Table 1).

### Contact Position Differences Among Functional Activities

**FIGURE 3:**
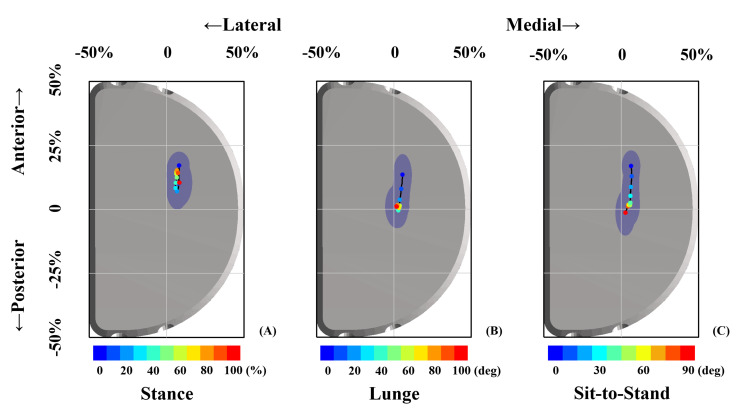
Articular contact excursions during the stance phase **(A)**, single-leg lunge **(B)**, and sit-to-stand motion **(C)** are shown. The scatters with different colors represented contact positions in the stance phase period **(A)** and knee flexion angle **(B,C)**. The blue dashed area indicated one standard deviation of contact position in anterior–posterior and medial–lateral directions.

FB UKA performed a significantly more anterior contact position with a smaller range of excursion during the stance phase than did those in deep lunge and sit-to-stand motions (Figure 3 and Table 1). The average anteroposterior contact position during the stance phase was 4.1 ± 2.0 mm anteriorly than lunge (*p* < 0.05) and 3.3 ± 3.1 mm anteriorly than sit-to-stand motion (*p* < 0.05). The effect power was 0.92. The range of an anteroposterior excursion during the stance phase was 1.9 ± 3.0 mm smaller than lunge (*p* < 0.05) and 2.6 ± 2.7 mm smaller than sit-to-stand motion (*p* < 0.05). The effect power was 0.87. No significant differences in average and range of an anteroposterior contact excursion were observed between lunge and sit-to-stand motions. Also, there were no significant differences in average and range of a mediolateral contact excursion among the stance phase, lunge, and sit-to-stand motion.

### Differences Between *in vivo* Contact Position and *in vitro* Wear Region

**FIGURE 4:**
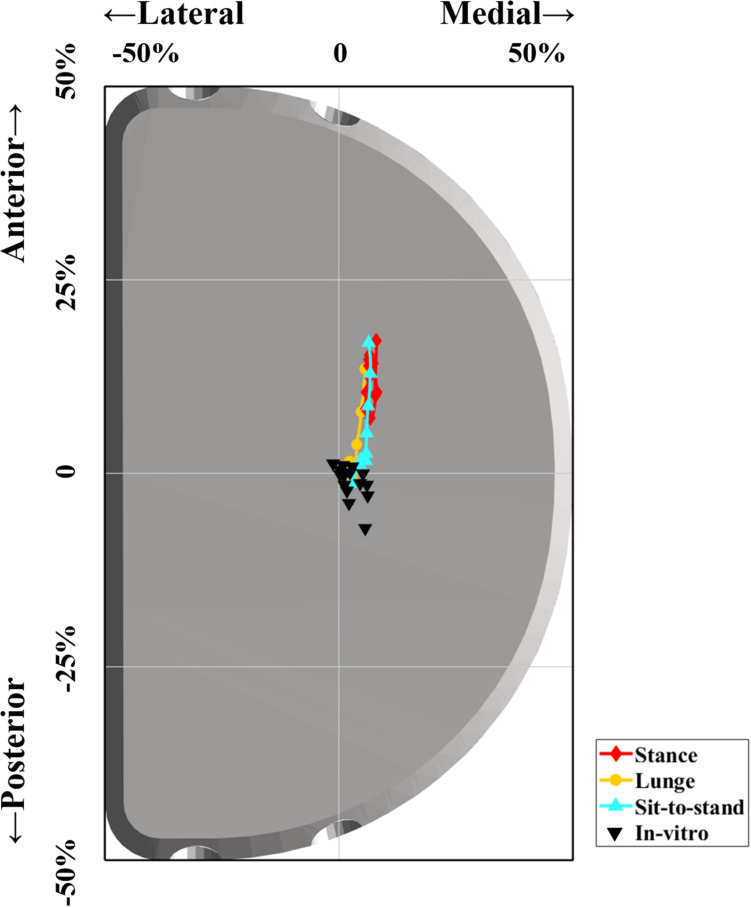
Difference between *in vivo* contact positions of FB unicompartmental knee arthroplasty (UKA) during functional activities and previous *in vitro* wear region center. The red, orange, and cyan lines indicate *in vivo* articular contact excursion during the stance phase, single-leg lunge, and sit-to-stand motion, respectively. The black triangles indicate the center of *in vitro* wear region or stress distribution area in previous studies (Kwon et al., 2013; Schroeder et al., 2013; Schwiesau et al., 2013b; Koh et al., 2019).

The average center of *in vitro* wear region or stress distribution area was 0.5 ± 1.0 mm posteriorly and 0.7 ± 0.6 mm medially after being normalized and mapped onto the current representative PE insert (Kwon et al., 2013; Schroeder et al., 2013; Schwiesau et al., 2013b; Koh et al., 2019). The *in vivo* contact positions located in a more anterior surface on PE insert of 5.7 ± 2.7 mm for the stance phase (*p* < 0.001), 1.5 ± 2.4 mm for lunge (*p* < 0.001), and 2.6 ± 3.3 mm for sit-to-stand motion (*p* < 0.001) than did *in vitro* wear region center. The *in vivo* contact positions located in a more medial surface on PE insert of 1.1 ± 1.6 mm for the stance phase (*p* < 0.001), 0.2 ± 1.5 mm for lunge (*p* = 0.14), and 0.7 ± 1.4 mm for sit-to-stand motion (*p* < 0.001) than did *in vitro* wear region center (Figure 4 and Table 1).

## Discussion

The current study quantified and compared the *in vivo* 6-DOF knee kinematics and articular contact positions during gait, single-leg lunge, and sit-to-stand activities in patients following medial FB UKA. We found that the 6-DOF kinematics and contact positions of FB UKA are different among different activities. The UKA contact was located anteriorly and medially on the PE insert and translated predominantly in the anteroposterior direction during different functional activities. A more anterior contact position and a smaller range of excursion were observed in the stance phase than in lunge and sit-to-stand motions. No significant differences were observed in a mediolateral contact position and range of excursion among the three investigated functional activities. The *in vivo* contact position in patients with FB UKA located in the more anterior part of the PE insert than the wear region of the *in vitro* wear simulator.

Accurate *in vivo* 6-DOF knee kinematics and articular contact behavior of the knee arthroplasty during functional activities are crucial factors to predict its biotribology and PE wear. However, only partial kinematic parameters of FB UKA have been reported, which is not sufficient in *in vitro* wear simulation (Mochizuki et al., 2013; Zumbrunn et al., 2020). In the current study, the specific *in vivo* 6-DOF showed different patterns between the stance phase during gait and weight-bearing knee flexion–extension (single-leg lunge and sit-to-stand) for the knee following FB UKA. It can be used as 6-DOF inputs in future knee wear simulation studies. Besides, limited studies analyzed the *in vivo* contact excursion of FB UKA. The anteroposterior contact position of the medial compartment remained relatively constant on the center of the superior surface during weight-bearing knee flexion in patients with posterior cruciate-retaining (CR) TKA (Suggs et al., 2009; Nicolet-Petersen et al., 2020). The medial articular contact position of bi-cruciate retaining (BCR) TKA translated posteriorly in the single stance phase of gait while moving anteriorly in the double stance phase (Tsai et al., 2019), and the contact excursion in the medial side from the center to posterior during deep lunge and sit-to-stand motions was observed in 29 unilateral BCR TKA patients (Arauz et al., 2019). Furthermore, the contact position moved around the center of the medial compartment in 15 healthy subjects during walking over the ground at self-selected speeds (Gray et al., 2019). In the current study, a more anterior articular contact position with a similar pattern and range of contact excursion in anteroposterior direction was observed for FB UKA patients compared with CR TKA, BCR, and TKA, and normal knees. The distinct contact patterns in FB UKA patients during different functional activities gave significant insights into the *in vivo* PE wear mechanism, indicating the importance of knee kinematics and contact tracking to predict the UKA failure during long-term follow-up.

The contact kinematics of medial FB UKA during the stance phase varied from lunge to sit-to-stand motions, indicating different biomechanical loading conditions in different functional motions. Level walking is the most common daily activity, and lunge and sit-to-stand motions represent the most challenging weight-bearing function with deep flexion angle for patients undergoing knee arthroplasty (Schwiesau et al., 2013a). The different contact kinematics of FB UKA in functional motions with the potential effect on PE insert wearing should be well-considered in implant design, preoperative testing, implant positioning, and postoperative rehabilitation for FB UKA patients to achieve better clinical outcomes.

The retrieved PE inserts showed different wear mechanisms in clinical conditions compared with the *in vitro* simulation results (Sutton et al., 2010; Schwiesau et al., 2013b). The central and medial wear region and the resultant volumetric wear of FB UKA after five million cyclic walking loading have been reported in knee simulator tests and finite element studies (Burton et al., 2012; Kwon et al., 2013; Schroeder et al., 2013). However, the articular contact excursion during a loading cycle was not quantified. In the current study, the contact positions were located in the more anterior part of the insert of FB UKA during the stance phase, lunge, and sit-to-stand motions than measured wear region in the knee simulator test and finite element study (Burton et al., 2012; Kwon et al., 2013; Schroeder et al., 2013). The anterior contact positions implied an increased probability of shearing on the superior surface of FB UKA PE insert under axial loading and anterior wear region under physiological activities. Furthermore, the level walking wear simulation test failed to reproduce the *in vivo* structural material fatigue and delamination mechanisms in PE gliding surfaces of FB UKA (Sutton et al., 2010; Schwiesau et al., 2013b). The increased relative motions, including rolling, gliding, and shearing in contact surfaces, were associated with a higher risk of delamination (Blunn et al., 1991), and the *in vivo* articular motion and delamination failure mode of FB UKA cannot be fully reproduced under force-control loading condition in the current *in vitro* knee wear simulator (Sutton et al., 2010; Schwiesau et al., 2013b). The realistic contact excursion on PE surface for *in vitro* knee wear simulator may replicate consistent wear region and failure mode with clinical outcomes, which contributes to implant design improvement.

Several factors can also result in different wear mechanisms between clinical conditions and *in vitro* simulation. The force and displacement loading during walking for FB UKA wear simulation are measured in TKA patients (Lu et al., 1998; Taylor et al., 1998), which are different from the *in vivo* situation for UKA patients (Jones et al., 2016). The incompletely replicated *in vivo* knee kinematics account for the differences in wear patterns between physiological conditions and *in vitro* studies (Sutton et al., 2010; Schwiesau et al., 2013b). Furthermore, the gravimetric wear of FB UKA is underestimated for level walking compared with high demanding activities, such as stair climbing, chair rising, and deep squatting (Schwiesau et al., 2013b), indicating the necessity to combine more weight-bearing functional activities for *in vitro* knee simulation. The articular contact excursion and relative kinematics of FB UKA with low congruency during the stance phase, single-leg lunge, and sit-to-stand were presented in the current study, which can be used as displacement-control criteria in FB UKA simulator tests and finite element studies to predict physiological wear performance for preclinical assessments. Besides, the PE is used on medial and lateral contact surfaces of the bi-compartment test device in an *in vitro* knee wear simulator. However, significantly different material properties between meniscus and PE for most patients undertaking single compartment UKA indicate different *in vivo* loading conditions than *in vitro* test (Kwon et al., 2013). A more anatomic test station with UKA mounted in a single compartment may better reproduce the *in vivo* knee kinematics and wear pattern.

Several potential limitations should be considered in the current study. First, only one type of FB UKA was investigated. However, the 6-DOF knee kinematics and contact excursion during functional activities can represent the *in vivo* contact of FB UKA with a low tibiofemoral congruency design. Other types of UKA should be studied in future work, such as MB UKA. Second, there was a lack of long-term follow-up data to explicitly verify the relationship between contact excursion and *in vivo* volumetric or linear wear rate of FB UKA. We will continue following up these UKA patients to track the long-term clinical outcomes and survival rate. Third, the contact stress distribution on the articular interface cannot be evaluated in the current study. A new device should be developed to measure internal force in the operated compartment to improve the evaluation of wear performance. Finally, only contact excursion on the superficial articular surface was quantified in the current study, and the inferior contact surface was not considered. However, the wear and deformation occur predominantly on the superficial interface in FB UKA. Only creep and minimal wear were observed in the inferior interface (Kretzer et al., 2011).

In conclusion, the current study quantified articular contact excursions of FB UKA during the weight-bearing stance phase of the gait cycle, single-leg lunge, and sit-to-stand motion. The contact position was located predominantly in the anterior and medial parts of the PE insert for all investigated functional activities, and a more anterior contact position and a smaller range of excursion were observed in the stance phase compared with lunge and sit-to-stand motions. The *in vivo* contact position of FB UKA during investigated activities was more anterior than the *in vitro* wear region, indicating that the actual internal knee kinematics of FB UKA should be well-reproduced for the knee simulator test and finite element study to improve the prediction of PE wear. The long-term *in vivo* wear performance and relative clinical outcomes should be tracked in the future study.

## Data Availability Statement

The original contributions presented in the study are included in the article/Supplementary Material, further inquiries can be directed to the corresponding author/s.

## Ethics Statement

The studies involving human participants were reviewed and approved by the Shanghai Sixth People’s Hospital. The patients/participants provided their written informed consent to participate in this study.

## Author Contributions

HD, NZ, QW, and T-YT contributed to the conception and design of study and participated in the writing of the manuscript. QW performed all surgeries. HD and NZ contributed to patient recruitment and data collection. HD, NZ, DZ, ZZ, and T-YT carried out the data analysis. HD, NZ, DZ, ZZ, ML, QW, and T-YT contributed to data interpretation. All authors gave approval of the final manuscript.

## Conflict of Interest

QW got research support from Stryker Corp., United States. T-YT got research support from MicroPort Co., Ltd., China. The remaining authors declare that the research was conducted in the absence of any commercial or financial relationships that could be construed as a potential conflict of interest.

## Footnotes

1 https://stats.idre.ucla.edu/other/gpower/

## Supplementary Material

Supplementary Figure 1 Average and standard deviation of 6-DOF kinematics of operated knee during stance phase of gait for unilateral FB UKA patients. **(A–C)** knee flexion/extension (F/E), tibial internal/external rotation (IR/ER), tibial adduction/abduction (ADD/ABD); **(D–F)** femoral anterior/posterior translation (A/P), proximal/distal translation (P/D) and medial/lateral translation (M/L). The rotations reported here described the tibial rotations relative to the femur. The translations represented the femoral motions relative to the tibia.

Supplementary Figure 2 Average and standard deviation of 6-DOF kinematics of operated knee during single-leg deep lunge for unilateral FB UKA patients. **(A–C)** knee flexion/extension (F/E), tibial internal/external rotation (IR/ER), tibial adduction/abduction (ADD/ABD); **(D–F)** femoral anterior/posterior translation (A/P), proximal/distal translation (P/D) and medial/lateral translation (M/L). The rotations reported here described the tibial rotations relative to the femur. The translations represented the femoral motions relative to the tibia.

Supplementary Figure 3 Average and standard deviation of 6-DOF kinematics of operated knee during sit-to-stand motion for unilateral FB UKA patients. **(A–C)** knee flexion/extension (F/E), tibial internal/external rotation (IR/ER), tibial adduction/abduction (ADD/ABD); **(D–F)** femoral anterior/posterior translation (A/P), proximal/distal translation (P/D) and medial/lateral translation (M/L). The rotations reported here described the tibial rotations relative to the femur. The translations represented the femoral motions relative to the tibia.
