# Supplementary figures and images for: More Anterior in vivo Contact Position in Patients With Fixed-Bearing Unicompartmental Knee Arthroplasty During Daily Activities Than in vitro Wear Simulator
Source: Front Bioeng Biotechnol. 2021 May 20;9:666435. doi: 10.3389/fbioe.2021.666435 (PMC8173134; doi:10.3389/fbioe.2021.666435)

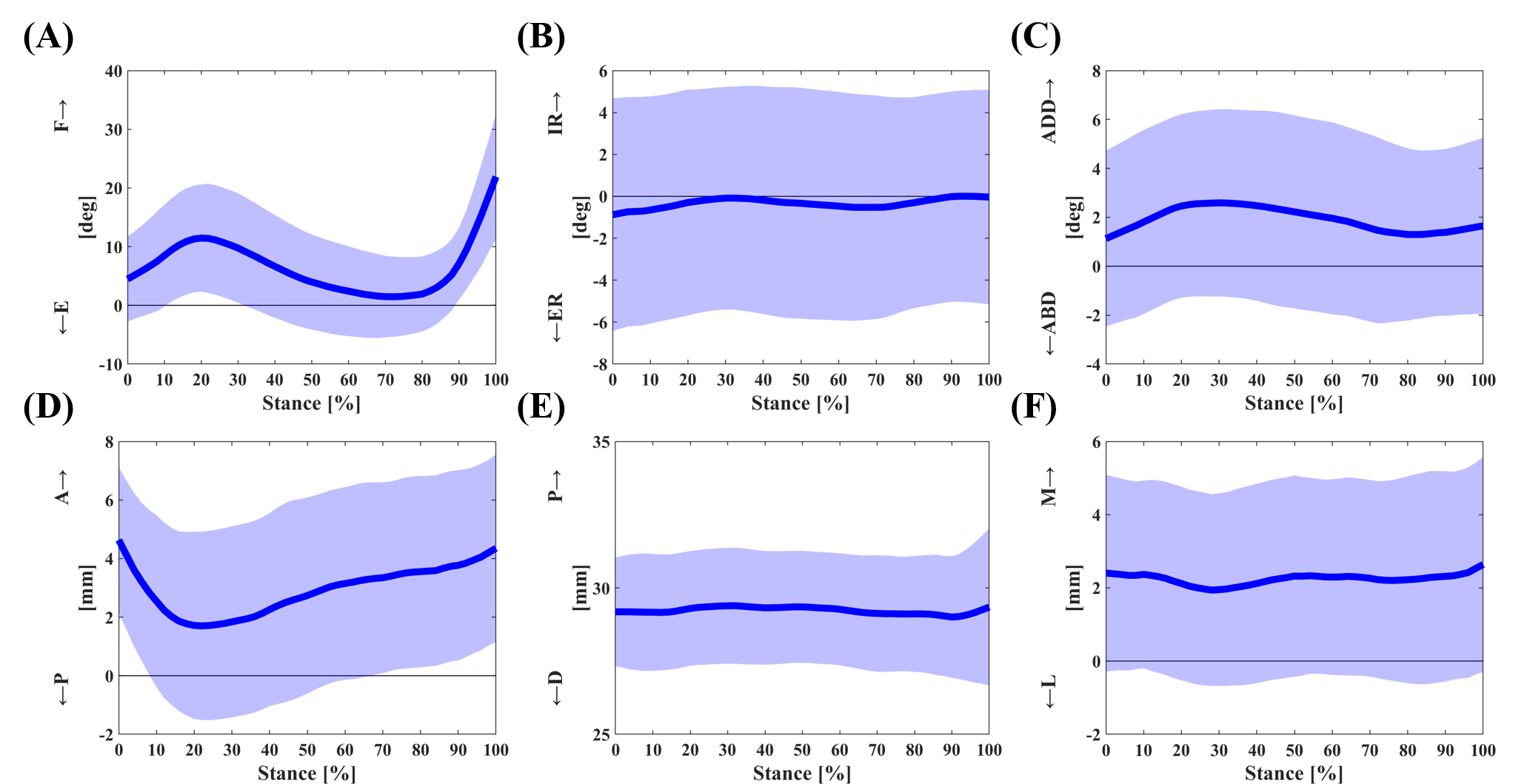

Supplement: Supplementary Figure 1 — Average and standard deviation of 6-DOF kinematics of operated knee during stance phase of gait for unilateral FB UKA patients. (A–C) knee flexion/extension (F/E), tibial internal/external rotation (IR/ER), tibial adduction/abduction (ADD/ABD); (D–F) femoral anterior/posterior translation (A/P), proximal/distal translation (P/D) and medial/lateral translation (M/L). The rotations reported here described the tibial rotations relative to the femur. The translations represented the femoral motions relative to the tibia. [file Image_1.TIF]

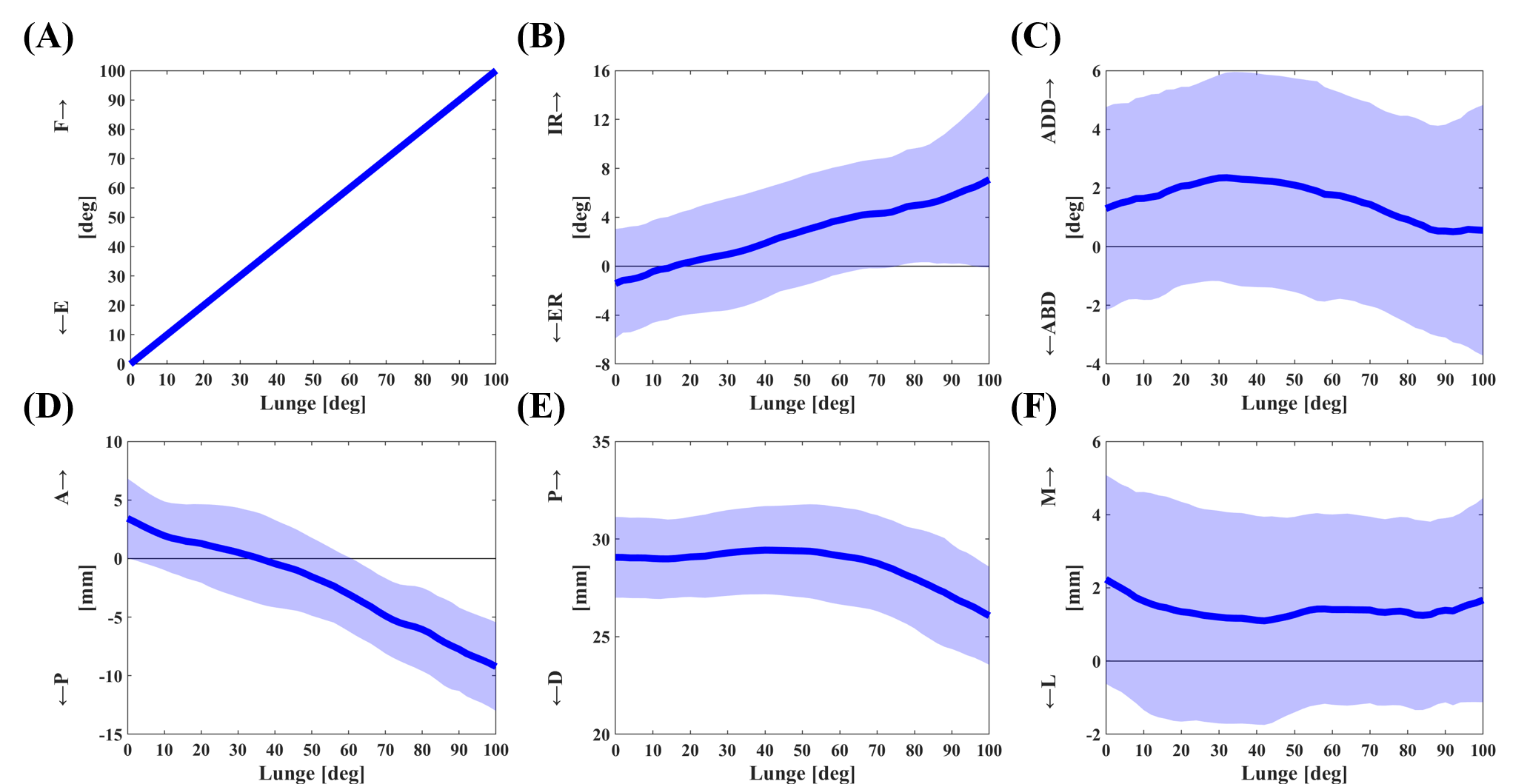

Supplement: Supplementary Figure 2 — Average and standard deviation of 6-DOF kinematics of operated knee during single-leg deep lunge for unilateral FB UKA patients. (A–C) knee flexion/extension (F/E), tibial internal/external rotation (IR/ER), tibial adduction/abduction (ADD/ABD); (D–F) femoral anterior/posterior translation (A/P), proximal/distal translation (P/D) and medial/lateral translation (M/L). The rotations reported here described the tibial rotations relative to the femur. The translations represented the femoral motions relative to the tibia. [file Image_2.TIF]

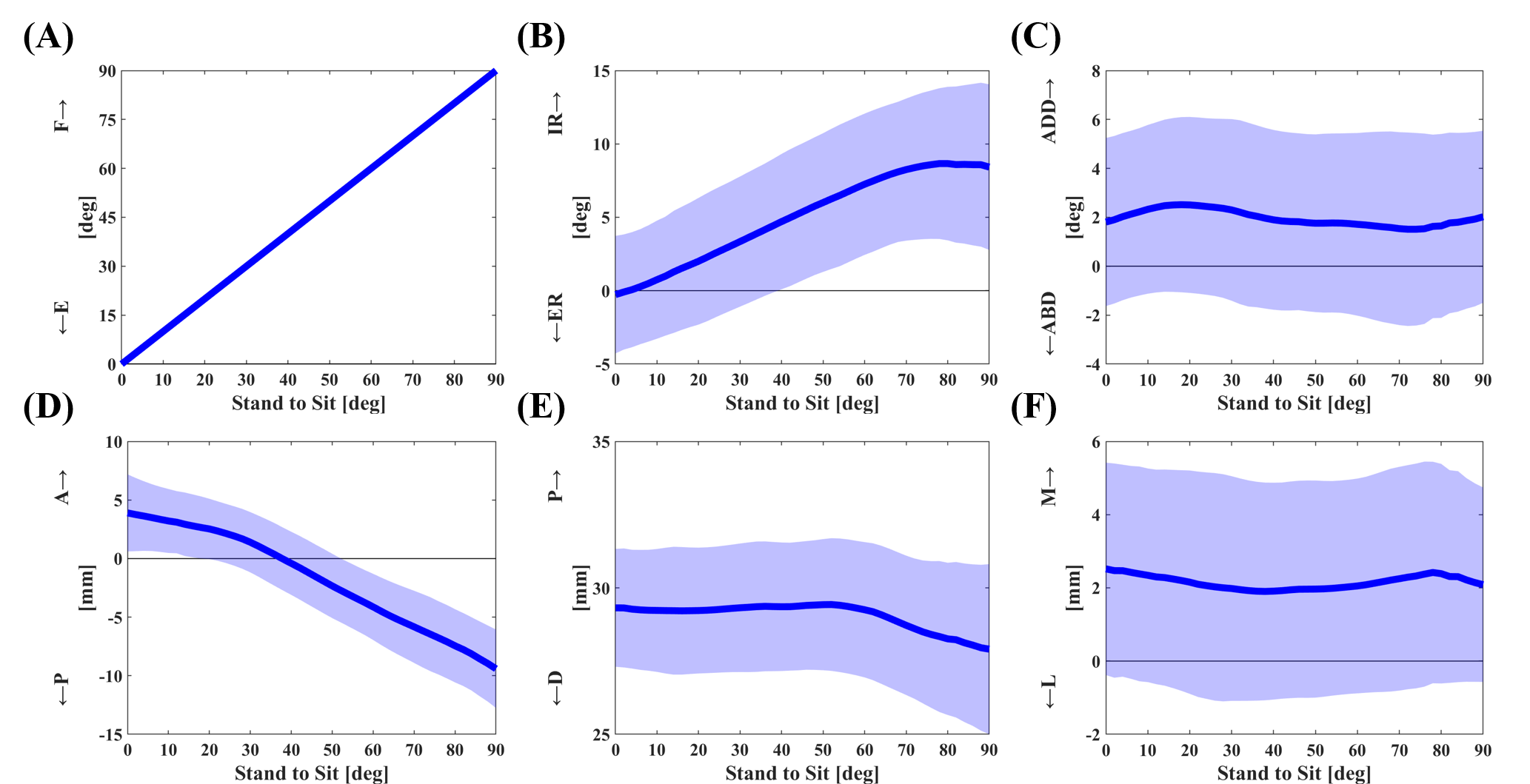

Supplement: Supplementary Figure 3 — Average and standard deviation of 6-DOF kinematics of operated knee during sit-to-stand motion for unilateral FB UKA patients. (A–C) knee flexion/extension (F/E), tibial internal/external rotation (IR/ER), tibial adduction/abduction (ADD/ABD); (D–F) femoral anterior/posterior translation (A/P), proximal/distal translation (P/D) and medial/lateral translation (M/L). The rotations reported here described the tibial rotations relative to the femur. The translations represented the femoral motions relative to the tibia. [file Image_3.TIF]
