# Supplementary material for: More Anterior in vivo Contact Position in Patients With Fixed-Bearing Unicompartmental Knee Arthroplasty During Daily Activities Than in vitro Wear Simulator
Source: Front Bioeng Biotechnol. 2021 May 20;9:666435. doi: 10.3389/fbioe.2021.666435 (PMC8173134; doi:10.3389/fbioe.2021.666435)
Supplement: Supplementary file 4 [file Table_1.docx]

Supplementary Table I. In-vivo contact position in anterior-posterior and medial-lateral directions during stance phase of gait cycle.

| **Stance Duration/%** | **Anterior-posterior** | |  | **Medial-lateral** | |
| --- | --- | --- | --- | --- | --- |
|  | **Average/mm** | **Normalized/%** |  | **Average/mm** | **Normalized/%** |
| **0** | 7.4±2.3 | 17.2±5.4 |  | 2.3±2.1 | 5.4±8.0 |
| **10** | 4.4±3.0 | 10.1±7.0 |  | 3.0±2.0 | 7.0±7.8 |
| **20** | 3.1±3.1 | 7.1±7.1 |  | 3.1±1.7 | 7.1±6.7 |
| **30** | 3.6±2.9 | 8.3±6.8 |  | 2.9±1.5 | 6.8±5.7 |
| **40** | 4.5±2.9 | 10.4±6.7 |  | 2.9±1.5 | 6.7±5.9 |
| **50** | 5.4±2.8 | 12.5±6.4 |  | 2.8±1.7 | 6.4±6.6 |
| **60** | 6.0±2.7 | 13.9±6.4 |  | 2.7±1.6 | 6.4±6.3 |
| **70** | 6.3±2.6 | 14.7±6.0 |  | 2.6±1.7 | 6.0±6.3 |
| **80** | 6.6±2.8 | 15.3±6.5 |  | 2.8±1.7 | 6.5±6.6 |
| **90** | 6.1±3.1 | 14.3±7.1 |  | 3.1±1.9 | 7.1±7.4 |
| **100** | 4.5±4.1 | 10.5±9.4 |  | 4.1±2.1 | 9.4±8.2 |

Data were given as average ± standard deviation
