# Supplementary material for: More Anterior in vivo Contact Position in Patients With Fixed-Bearing Unicompartmental Knee Arthroplasty During Daily Activities Than in vitro Wear Simulator
Source: Front Bioeng Biotechnol. 2021 May 20;9:666435. doi: 10.3389/fbioe.2021.666435 (PMC8173134; doi:10.3389/fbioe.2021.666435)
Supplement: Supplementary file 5 [file Table_2.docx]

Supplementary Table II. In-vivo contact position in anterior-posterior and medial-lateral directions during the single-leg lunge.

| **Knee Flexion/**° | **Anterior-posterior** | |  | **Medial-lateral** | |
| --- | --- | --- | --- | --- | --- |
|  | **Average/mm** | **Normalized/%** |  | **Average/mm** | **Normalized/%** |
| **0** | 5.8±3.5 | 13.5±8.0 |  | 3.5±1.4 | 8.0±5.6 |
| **10** | 3.4±3.5 | 8.0±8.2 |  | 3.5±1.2 | 8.2±4.8 |
| **20** | 1.6±3.2 | 3.7±7.4 |  | 3.2±1.0 | 7.4±3.8 |
| **30** | 0.1±3.1 | 0.3±7.2 |  | 3.1±0.8 | 7.2±3.1 |
| **40** | -0.2±2.4 | -0.4±5.5 |  | 2.4±0.7 | 5.5±2.9 |
| **50** | 0.3±2.1 | 0.7±4.8 |  | 2.1±0.9 | 4.8±3.5 |
| **60** | 0.6±2.2 | 1.4±5.1 |  | 2.2±1.0 | 5.1±3.7 |
| **70** | 0.2±2.9 | 0.5±6.6 |  | 2.9±0.8 | 6.6±3.2 |
| **80** | 0.7±3.6 | 1.6±8.3 |  | 3.6±0.6 | 8.3±2.3 |
| **90** | 0.4±3.4 | 0.9±7.9 |  | 3.4±0.5 | 7.9±1.9 |
| **100** | 0.5±3.6 | 1.1±8.4 |  | 3.6±0.4 | 8.4±1.7 |

Data were given as average ± standard deviation
