# Supplementary material for: More Anterior in vivo Contact Position in Patients With Fixed-Bearing Unicompartmental Knee Arthroplasty During Daily Activities Than in vitro Wear Simulator
Source: Front Bioeng Biotechnol. 2021 May 20;9:666435. doi: 10.3389/fbioe.2021.666435 (PMC8173134; doi:10.3389/fbioe.2021.666435)
Supplement: Supplementary file 6 [file Table_3.docx]

Supplementary Table III. In-vivo contact position in anterior-posterior and medial-lateral directions during sit-to-stand motion.

| **Knee Flexion/**° | **Anterior-posterior** | |  | **Medial-lateral** | |
| --- | --- | --- | --- | --- | --- |
|  | **Average/mm** | **Normalized/%** |  | **Average/mm** | **Normalized/%** |
| **0** | 7.3±2.6 | 16.9±6.1 |  | 2.6±1.7 | 6.1±6.4 |
| **10** | 5.5±3.3 | 12.9±7.6 |  | 3.3±1.8 | 7.6±6.7 |
| **20** | 3.7±3.9 | 8.7±9.2 |  | 3.9±1.6 | 9.2±6.2 |
| **30** | 2.2±4.1 | 5.2±9.6 |  | 4.1±1.5 | 9.6±6.0 |
| **40** | 1.1±3.8 | 2.5±8.8 |  | 3.8±1.5 | 8.8±5.9 |
| **50** | 0.7±3.4 | 1.6±8.0 |  | 3.4±1.5 | 8.0±5.7 |
| **60** | 0.7±3.2 | 1.6±7.4 |  | 3.2±1.3 | 7.4±5.1 |
| **70** | 0.7±3.1 | 1.7±7.2 |  | 3.1±1.2 | 7.2±4.7 |
| **80** | 0.5±3.3 | 1.1±7.6 |  | 3.3±1.2 | 7.6±4.4 |
| **90** | -0.6±3.9 | -1.4±9.0 |  | 3.9±0.7 | 9.0±2.7 |

Data were given as average ± standard deviation
